# Subsite-specific contributions of different aromatic residues in the active site architecture of glycoside hydrolase family 12

**DOI:** 10.1038/srep18357

**Published:** 2015-12-16

**Authors:** Xiaomei Zhang, Shuai Wang, Xiuyun Wu, Shijia Liu, Dandan Li, Hao Xu, Peiji Gao, Guanjun Chen, Lushan Wang

**Affiliations:** 1 The State Key Laboratory of Microbial Technology, Shandong University, Jinan, 250100, P.R. China; 2 Key Laboratory of Colloid and Interface Chemistry, Ministry of Education, Institute of Theoretical Chemistry, Shandong University, Jinan, 250100, P.R. China

## Abstract

The active site architecture of glycoside hydrolase (GH) is a contiguous subregion of the enzyme constituted by residues clustered in the three-dimensional space, recognizing the monomeric unit of ligand through hydrogen bonds and hydrophobic interactions. Mutations of the key residues in the active site architecture of the GH12 family exerted different impacts on catalytic efficiency. Binding affinities between the aromatic amino acids and carbohydrate rings were quantitatively determined by isothermal titration calorimetry (ITC) and the quantum mechanical (QM) method, showing that the binding capacity order of Tyr>Trp>His (and Phe) was determined by their side-chain properties. The results also revealed that the binding constant of a certain residue remained unchanged when altering its location, while the catalytic efficiency changed dramatically. Increased binding affinity at a relatively distant subsite, such as the mutant of W7Y at the −4 subsite, resulted in a marked increase in the intermediate product of cellotetraose and enhanced the reactivity of endoglucanase by 144%; while tighter binding near the catalytic center, i.e. W22Y at the −2 subsite, enabled the enzyme to bind and hydrolyze smaller oligosaccharides. Clarification of the specific roles of the aromatics at different subsites may pave the way for a more rational design of GHs.

Lignocellulose is the most abundant and sustainable resource and its conversion to biofuels is a highly desirable approach to fuelling the energy-exhausted earth^1^,^2^. The degradation of lignocellulose requires the effective synergy of different Carbohydrate-active enzymes (CAZymes), and concerted efforts have been made to produce new enzymes with improved catalytic efficiency^3^,^4^. Glycoside hydrolases (GHs) are a group of enzymes which hydrolyze glycosidic bonds and include more than 100 sequence-based families in the CAZy database (http://afmb.cnrs-mrs.fr/CAZY/)^5^. As a model for studying cellulose degradation mechanisms, the ascomycete fungus *Trichoderma reesei* (syn. *Hypocrea jecorina*) has already been extensively used in industry^6^. Whole genome sequencing of *T. reesei* showed that there were 200 genes encoding GH and 10 of these genes have been biochemically determined to be well-known cellulases, including cellobiohydrolase (CBH)I (Cel7A), CBHII (Cel6A), endoglucanase (EG)I (Cel7B), EGII (Cel5A and Cel5B), EGIII (Cel12A), EGIV (Cel61A, Cel61B and Cel74A) and EGV(Cel45A) from 7 different GH families^7^,^8^.

Different GH families have been proved to have low sequence similarities with each other, therefore they possess different active-site architectures and various enzymatic functions^9^. The active site architecture of GH is a contiguous subregion consisting of spatially clustered residues, and plays significant roles in substrate recognition, catalysis, product release and processivity^10^,^11^. There have been numerous well-established sequence- and structure-guided strategies for studying active site architecture^12^,^13^. Sequence logo, for instance, is a very powerful tool for identifying key residues at different binding subsites, thereby creating a “smaller but smarter” candidate library for rational design^14^,^15^. When combining the above-stated bioinformatics approaches with experimental tests, previous studies had already illustrated the functions of some active-site amino acids in detail. A typical example is a member of the GH5 family, Cel5B_Dtu, which obtained dual-substrate specificity and catalytic efficiency enhancement after the mutation of a polar residue (D14) formed hydrogen bonds with a ligand in the active site architecture^16^. Furthermore, catalytic efficiency enhancement was more likely to be attributed to the increase in the binding affinity constant (*k*_on_) rather than the more stable catalytic constant (*k*_cat_)^17^.

With regard to the binding affinity contributors, hydrogen bonds and hydrophobic interactions are the two main sources for GH. The strength of the polar, short-range, more specific H-bond was proved to be related to its distance and angle, while that of the fluid-like hydrophobic stacking was not fully clarified. Although studies relevant to the aromatics are prevalent, changes caused by aromatic amino acid mutations in both the free binding energy and the catalytic efficiency varied greatly^18^,^19^,^20^. Consequently, there are still no common rules on engineering GH through aromatic amino acid manipulations^21^. Further research is necessary.

The GH12 family is a widely-distributed member of the GH Clan-C, which adopts a typical β-sandwich fold and an inverting catalytic mechanism^8^,^22^. Up to Jan 1st 2015, there were 367 sequences, with 52 characterized members, 13 resolved structures and 34 entries deposited in the RCSB Protein Data Bank (PDB, http://www.rcsb.org/pdb/home/home.do). Previous studies have revealed that GH12 enzymes exhibited several interesting characteristics including being induced at the initial stage with the smallest size (around 20 kDa), lacking a carbohydrate-binding module (CBM), multifunction, and having a wide range of optimal pH values ([pH_opt_]) as well as temperature values for growth ([T_opt_])^23^,^24^,^25^. The diversity of properties in the GH12 family makes it as an ideal candidate for studying specific contributions of residues to enzymatic functions.

To gain further insights into factors influencing the hydrophobic interactions, the GH12 family was selected for research, and a bioinformatic analysis as well as biochemical determinations were carried out. ITC and QM results showed that the binding capacities of aromatic residues were determined by their side-chain properties in the following order: Tyr>Trp>His (and Phe). Binding constants of the aromatics remained approximately the same when their location was altered, however, the catalytic efficiency and the mode of action of the enzyme changed dramatically. It is hoped that the key findings in this study will have wide applications in guiding the rational design of other more divergent GH families.

## Results

### Constructing a sequence logo of the GH12 family

**Figure 1:**
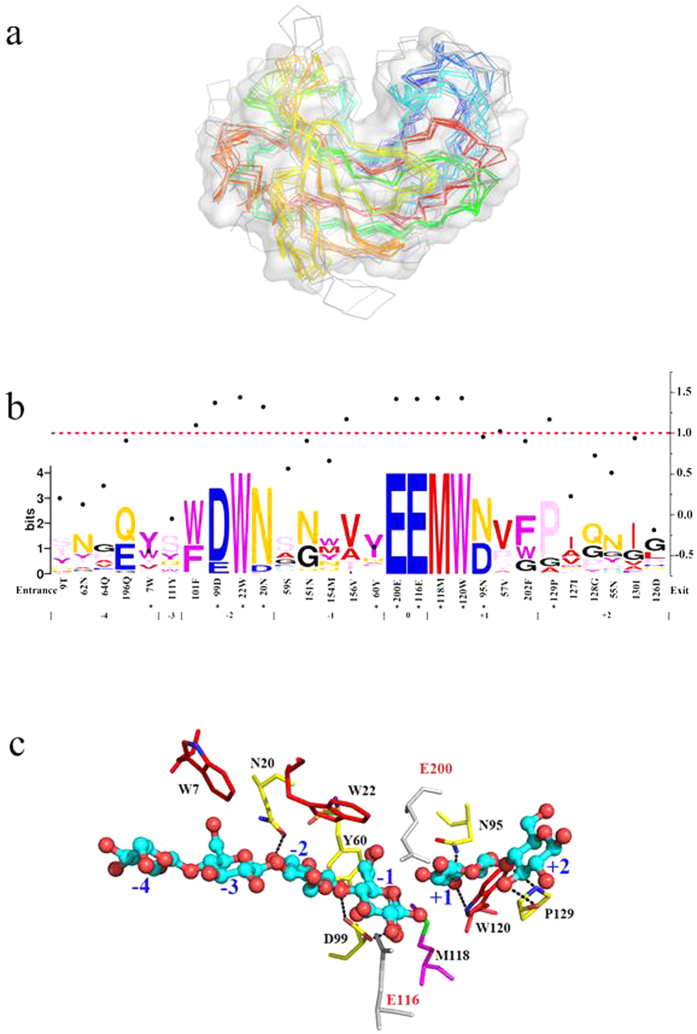
Structure alignment of the GH12 family and conservation degree evaluation of the active-site sequence profile. (**a**) Superimposition of GH12 members with PDB ID. Surface of *Tr*Cel12A (taken as reference) was displayed in white with the catalytic cleft in the middle. Other 3D structural ligands and alignment to *Tr*Cel12A were performed and the results of the RMSD were listed in Table S1. (**b**) Active-site sequence profile of GH12 family and the conservation degree evaluation of each site. Residues determinant to the catalytic efficiency were labeled in asterisk (*) and the numbers seperated below were the binding subsites. The conservation degree of each residue, which was marked with a corresponding bold dot, was evaluated by the minus value of the normalized ConSurf Score calculated at the website http://consurf.tau.ac.il/index_proteins.php. The dividing dash line of 1.0 was marked in red. (**c**) Mapping of the key residues in the active site of *Tr*Cel12A. The cellohexaose docked in the binding crevice was taken from *Bl*Cel12A for similarity of the two structures. The catalytic residues and the aromatics were colored gray and red, while the H-bonds were shown by the dotted line with the donors colored in yellow.

To accurately identify the function-determinant residues, an active site architecture sequence profile of the GH12 family was constructed. The pair-wise structure alignment to 1H8V showed that the typical endoglucanase GH12 members exhibited an overall conservative architecture of the β-jelly roll (Fig. 1a) with low RMSD values (all below 1.11 Å except those of 3AMH and 3VGI, Table S1). The active site was a large crevice 35 Å long, which could accommodate six glucose-binding subsites (namely −4 to + 2 from the nonreducing end to the reducing end). Residues lying within 5 Å to the binding oligosaccharide at each site were selected and the sequence profile, taking *Tr*Cel12A as the reference, is shown in Fig. 1b.

### Analysis of the sequence logo

As seen in Fig. 1b, there were five residues with absolute conservation across the GH12 family. In *Tr*Cel12A, they corresponded to W22, M118, W120 and the catalytic residues E116 and E200, which clustered together on the −2 to the +1 subsite. In contrast, residues at the −4, −3 and +2 subsites were variable. For example, residue 7 at the −4 subsite could be Tyr, Trp or Phe etc. From the combined results of the evolutionary conservation degree of each site in *Tr*Cel12A, it can be seen that the scores of most residues in the −2 to +1 subsites were relatively high, while those of the distant residues were low, which further proved that “the nearer the residues are to the catalytic center, the higher conservation degree they have^26^”. In addition, it was confirmed that residues substitutable at the top of the heap with the largest proportional symbol size at a given site were more likely to appear in the long evolutionary history, whereas those at the bottom with a relatively small size were more likely to be replaced. For instance, the odds of tyrosine appearing at residue 7 were 38%, while that of tryptophan were just 17% as revealed by multiple sequence alignment.

Moreover, closer examination of the *Tr*Cel12A complex (Fig. 1c) revealed that glucosyl at the −2 subsite was oriented directly towards the conservative aromatic residue Trp22, which helps to stabilize sugars by hydrophobic stacking interactions^27^. Two additional residues with a high degree of conservation, Asn20 and Asp99, also formed hydrogen bonds at O2 and O4 of the glucose at this subsite, respectively. Furthermore, glucose moieties in the −1 and +1 subsites were anchored by several hydrogen bond providers. With the exception of the two catalytic residues at the β-1, 4-linkage, hydrogen bonds can also be formed by Tyr60 at C6 and Asn151 at C2 and C3 hydroxyls of the glucose at the −1 subsite, while the three highly conservative residues Met118, Asn95 and Trp120 participated in hydrogen bond formation at O4, O3 and O3 of the glucose at the +1 subsite.

### Tracing key residues in the active site architecture of *Tr*Cel12A

**Figure 2:**
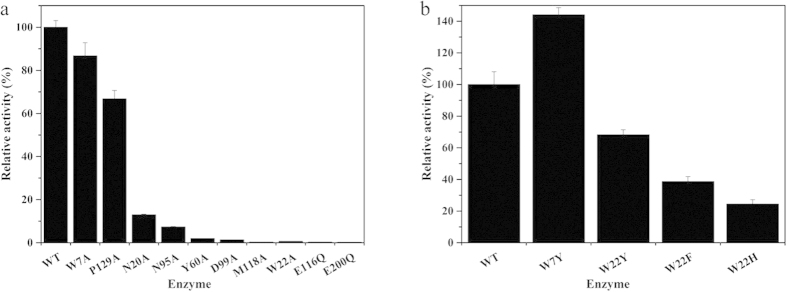
Relative endoglucanase activities. Relative activities of the alanine mutations constructed (**a**), except E116Q and E200Q) and (**b**) the structurally related to the two specific tryptophans in the active site architecture of *Tr*Cel12A. Data are the means of three independent results; error bars show s.d.

To detect the relative importance of key residues in the active site architecture, alanine scanning of *Tr*Cel12A (except E116Q and E200Q) was first conducted. Comparison of the CD spectra revealed that there was almost no difference between the constructed mutants and the WT enzyme (Fig. S1), indicating mutations didn’t affect the conformation or stability of *Tr*Cel12A. Endoglucanase activities (Fig. 2a) have shown that mutations of the highly-conserved residues at the −2 to +1 binding subsites, for example, N20A, W22A, N95A, D99A and M118A showed significant reduction. Alanine substitutions of M118 at the −1 subsite and W22, D99 at the −2 subsite had virtually no activity with less than 1% of the WT left, indicating their essential roles in catalysis. In addition, mutants of *Tr*Cel12A^Y60A^ at the −1 subsite, *Tr*Cel12A^N95A^ at the +1 subsite and *Tr*Cel12A^N20A^ at the −2 subsite retained 1.60%, 7.31% and 13.0% of the WT endoglucanase. However, the alanine substitutions of residues relatively distant from the catalytic center did not have much effect. For example, the endoglucanase activities of P129A at the subsite of +2 and W7A at the subsite of −4 were only reduced by 33.2% and 13.3%. Overall, residues at a certain locus with diverse side-chain properties or those with the same side chain but at different subsites, for example, tryptophan at the subsites of −2 and −4, i.e. W22 and W7, exerted different impacts on enzymatic activities.

To gain further insight into influencing factors, the structurally-guided mutations of W22Y, W22F, W22H and W7Y were constructed. Results of the relative endoglucanase activities (Fig. 2b) revealed that the W7Y mutant at the −4 subsite enhanced catalytic efficiency by 144%, while the activity of W22Y decreased to 68.3%. Furthermore, larger losses in the activities resulting from mutations at the subsite of −2 were observed, and W22F and W22H retained only 38.7% and 24.5% of the WT endoglucanase, respectively.

### Monitoring thermodynamic parameter changes from mutations of key residues

**Figure 3:**
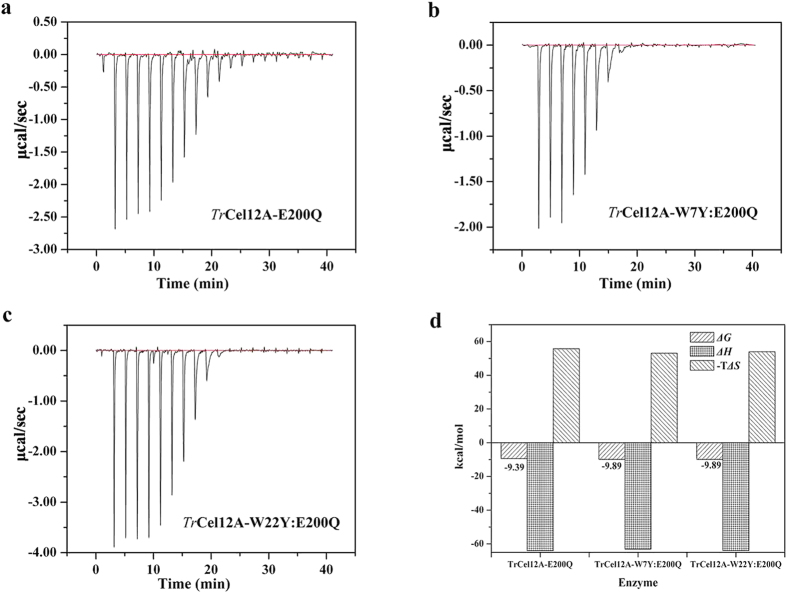
Isothermal titration calorimetry and thermodynamic parameters determined. The raw binding heat levels of (**a**) *Tr*Cel12A-E200Q with the concentration of protein 10.6 μM and that of ligand 22.0 μM, (**b**) *Tr*Cel12A-W7Y:E200Q with [protein] = 6.40 μM, [ligand] = 22.0 μM and (**c**) *Tr*Cel12A-W22Y:E200Q with [protein] = 11.5 μM, [ligand] = 27.5 μM.

Double mutations of W22 and W7 were further conducted on the basis of the inactivated mutant E200Q, and ITC was used to monitor changes in the thermodynamic parameters. It can be seen from Fig. 3a,b,c that binding of the GH12 family to the CMC was spontaneous with Gibbs free energy changes (Δ*G*) below zero. Comparison of the main thermodynamic parameters (Fig. 3d) has shown that Δ*G* values of the tyrosine mutants became more negative than that of the WT (−9.89  kcal/mol *vs.* −9.39 kcal/mol), leading the association constant (*K*_*a*_) of the former (1.91 × 10^7^ M^−1^) larger than the latter (8.18 × 10^6^ M^−1^). Also, it was shown from Fig. 3d that, although values of Δ*H* were almost the same (about −63.7 kcal/mol), the product of −TΔS decreased from tryptophan (55.7 kcal/mol) to tyrosine (53.5 kcal/mol), indicating that either the solvation entropy or the conformational entropy changed after mutation. Overall, analysis above revealed that tyrosine was more favorable in substrate binding regardless of where it was located.

The thermodynamic parameters of W22H, W22F and W22A were also determined. The results (Fig. S2) showed that Δ*G* values were −9.23, −9.21 and −8.88 kcal/mol, respectively, resulting in nearly the same association constant of W22H and W22F (6.27 × 10^6^ M^−1^ *vs.* 6.04 × 10^6^ M^−1^), while that of W22A decreased to 3.45 × 10^6^ M^−1^. Sharp decrease of the binding capacity of the W22A mutant also indicated that the carbohydrate-ring stacking interaction at the −2 subsite played a critical role in substrate binding.

### Determining the influence of the side-chain properties on binding energy

The interaction energy of the mutations of W22 and W7 to glucose was theoretically determined by the QM method. As there was no difference between the main chains of *Tr*Cel12A and its mutants (structured in peptide bonds), to reduce computing complexity^28^,^29^,^30^, models of the binding glucose moiety and the side chains of tryptophan, tyrosine, histidine, phenylalanine and alanine (i.e. indole, phenol, imidazole, benzene and methane, respectively) were selected. The structure and geometry of these groups were optimized and the optimal configuration of indole, imidazole and benzene was parallel with the glucose ring, supporting the formation of the C-H…π interactions. However, with regard to phenol, the side-chain hydroxyl group skewed significantly in the direction of the C2 of glucose, forming a new hydrogen bond, but weakened the carbohydrate-ring stacking interactions at the same time. Consequently, the theoretical interaction energy was calculated to be 11.47, 12.29, 7.49, 7.46 and 1.42 kcal/mol, respectively. The optimal geometry of the side-chain group was finalized when the interaction energy for glucose reached the minima of the local, and it was then inferred that interaction energies of the complex were closely related to properties of the moieties and not their spatial position.

**Figure 4:**
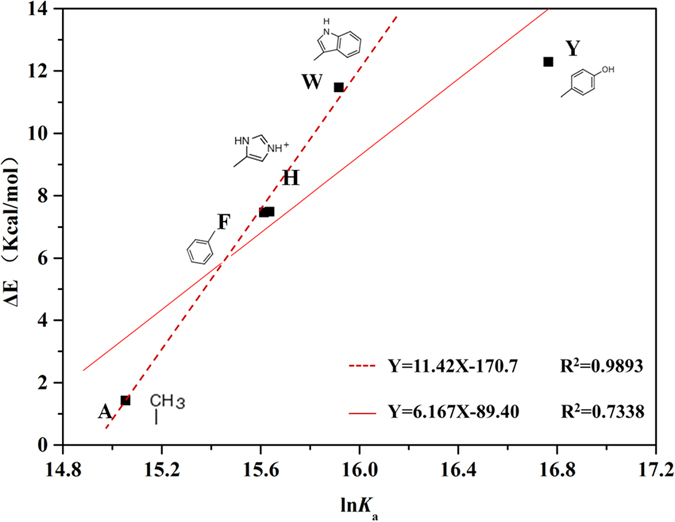
Correlation analysis of the theoretical interaction energy determined by QM calculations (Δ*E*) and the logarithm of the adsorbing constant from ITC (ln*K*_a_). Labels mapped on the figure are side chains of alanine (A), phenylalanine (F), histidine (H), tryptophan (W) and tyrosine (Y) from the lower left to the upper right, respectively. The solid fitting line involves all the mutations constructed, while, the dashed one is merely concerned with relevant correlations of A, F, H and W.

Not only that, but it was proved that there was a good linear relationship between the values of Δ*E* and the logarithm of the absorbing constant determined by ITC (ln*K*_*a*_) with a correlation coefficient (R^2^) of 0.734 (Fig. 4), indicating that the order of binding energy was Tyr>Trp>His (and Phe)>Ala. Furthermore, when considering only the binding affinities of Trp, His, Phe and even Ala, the Δ*E* values seemed to have a stronger correlation with ln*K*_*a*_ with the equation of Y = 11.42X −170.7 and a higher R^2^ value of 0.989, confirming that the results of the two methods were consistent and the low R^2^ value of the former was probably due to the sharp increase of the binding energy of tyrosine.

### Exploring the specific subsite contributions to catalytic efficiency

To obtain an in-depth understanding of the relationship between substrate binding and catalytic efficiency, kinetic parameters of the CMC were obtained and the results are summarized in Table 1. The K_m_ values of the tyrosine mutations at both the −4 and the −2 subsites decreased by almost the same extent, indicating that both values of the adsorbing constant (1/K_m_) increased by almost 2-fold compared with the WT. However, the values of *k*_cat_ varied significantly. Compared with the WT, the product release rate of W22Y was decreased by approximately 4-fold, while that of W7Y was less than 2-fold. Consequently, the *k*_cat_/K_m_ values differed. For W7Y, the strengthened binding affinity offset the small decline in the turnover number, leading to a 114% increase in endoglucanase, whereas W22Y retained only 44.9% due to tighter binding and delayed release of product. Enhancement of the subsite-binding energy at different sites resulted in distinct differences in enzymatic activity. Thus, enzymatic efficiency was subsite determined. Higher binding capacity does not necessarily guarantee improved specificity, and higher catalytic efficiency requires a good balance between substrate binding and product release.

The kinetic parameters of W22F and W22H were also determined and the results are shown in Table S2. It can be seen that the adsorbing constants of W22F and W22H were 85.9% and 52.6% of the WT and the corresponding *k*_cat_ values decreased to 34.0% and 49.6%, respectively. Thus, W22F and W22H retained 29.2% and 26.1% of endoglucanase activity, respectively. It should be mentioned that a good linear relationship was observed between the catalytic efficiency calculated by the Michaelis-Menten equation and the relative activity measured in Fig. 2b, with the equation Y = 0.965X −5.80 and an R^2^ value of 0.889.

### Probing the probable transformation of the mode of action due to mutations at the two specific subsites

**Figure 5:**
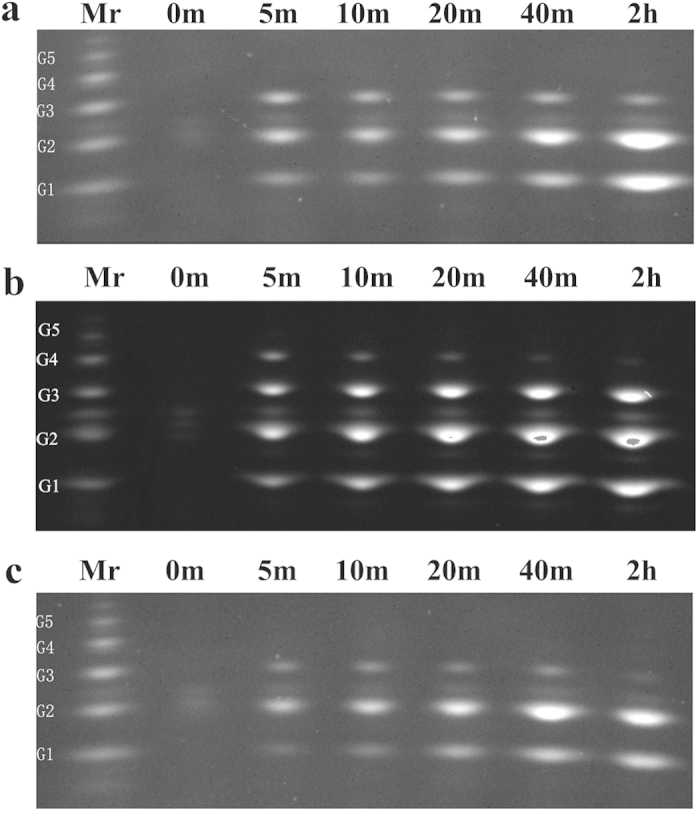
Time courses of the product profiles of TrCel12A and the tyrosine substitutions on PASC. (**a**) WT, (**b**) W7Y and (**c**) W22Y. Time intervals are marked on the top of each lane and abbreviations of the sugar in the marker (Mr) listed on the left are: G1-glucose, G2-cellobiose, G3-cellotriose, G4-cellotetraose and G5-cellopentose.

To further explore the reasons behind the specific subsite contributions to catalytic efficiency, FACE was used in the time-course analysis of the hydrolytic products on PASC. As shown in Fig. 5, there were significant differences in both the types and the amounts of oligosaccharides produced, which was caused by a single mutation of the key residues in the active site architecture. Similar to the profile of the WT (Fig. 5a), glucose (G1), cellobiose (G2), cellotriose (G3) and trace cellotetrose (G4) coexisted in the mutant W7Y hydrolysate in the initial stage of hydrolysis (Fig. 5b). With time, the concentration of cellotetrose showed a downward trend after the first increase (within 5 min), while that in the WT was almost undetectable. This indicated that G4 was first derivatized as the product, with the amount produced by W7Y larger than that of the WT, and then was further hydrolyzed to G1, G2 and G3. Gradual degradation of G3 was observed after a long period of time (e.g., 2 h or longer), and this indicated that G3 was not as good as G4, and G4 may be the minimum binding unit for both WT and W7Y.

The above consistency in the hydrolytic pattern verified that changes in the subsite-binding energy at the relatively distant subsite had almost no effect on both substrate specificity and enzymatic function. In contrast, the changes in the more centered subsite of −2, such as W22Y, and the distribution of the degradation products were altered. It can be seen from Fig. 5c that there was almost no G4 detected and G3 was more transient. The difference in product distribution can be explained by the fact that the W22Y mutant also produced all soluble oligosaccharides, but had a greater capacity to bind and hydrolyze cellotetrose and cellotriose.

It was reported previously that the most striking feature of different types of endoglucanase was their enzymatic specificity for amorphous cellulose with various degrees of polymerization (DPs)^31^. The improved binding and catalytic efficiency to G3 further indicated that the mode of action of W22Y has been shifted from one type of endoglucanase to another, proving that the centered residue of W22 played a direct role in substrate specificity. In addition, the binding and catalytic efficiency of W22H and W22F were weakened as illustrated above, and the intermediate product of G4 (and even G3) was also proved to be able to persist longer, while almost no G1 was observed in the initial stage (Fig. S3). The production of more soluble carbon sources other than monosaccharide detected in the hydrolytic profiles of W22H and W22F provided an ideal alternative for the effective induction of the lignocellulose-degrading enzymes^21^. Overall, analysis of the hydrolytic pattern revealed that key aromatic residues at different loci in the active site architecture may result in different impacts on substrate specificity as well as the enzymatic mode of action^31^.

## Discussion

The GH12 family has a long evolutionary history with members widely distributed in the kingdoms of bacteria, archaea and eukaryota (shadowed in blue, yellow and pink, respectively, Fig. S4). The T_opt_ value of certain members, especially those from the hyperthermophilic archaeon, can even reach as high as 100 °C^32^,^33^. Also, the range of pH_opt_ is extraordinarily wide, with values ranging from pH 1.8 to pH 8.0^34^,^35^. Not only that, but the GH12 family is multi-functional. With the exception of the majority of endoglucanases, it also has activities on xyloglucan, β-1,3-1,4-glucan and xylan, which are labeled in the red branches. These features of the GH12 family have powerful industrial application and are worthy of further improvement^36^.

The endoglucanase activity changes of the alanine mutations of *Tr*Cel12A, especially that of the W22A at the −2 subsite (Fig. 2a) illustrated that the sequence logo facilitated approach has great potential in tracing key residues of the GH family. Importance of the tryptophan at the −2 subsite was further supported by the mutational results of another member of the GH12 family, *An*EglA. Determination of the endoglucanase activities of *An*EglA and its mutants to W24 (corresponding to W22 at the −2 subsite in *Tr*Cel12A) has revealed that, the extent of the enzymatic activity loss was almost equal. Substitutions of the tryptophan by tyrosine, phenylalanine, histidine and alanine maintained 52.5%, 26.4%, 18.9% and 0.53% of the WT (Fig. S5a), while that in *Tr*Cel12A was 68.3%, 38.7%, 24.5% and 0.62%, respectively (Fig. 2). Not only that, but there was a good linear relationship between the relative activities of *An*EglA and those of *Tr*Cel12A, with a considerable R^2^ value of 0.9909 (Fig. S5b). Besides, multi-structure alignment of the PDB structures of GH12 members has revealed that the conformation of the indole ring at the −2 subsite was quite stable (Fig. S6). Thereby, it is reasonable to infer that, the tryptophan at the −2 subsite was both sequence and structure-conservative, and functionally equivalent in the whole GH12 family. Unlike the tryptophan at the −2 subsite which has absolute conservation, residues interacting with the glucosyl at the −4 subsite, including Trp7, were relatively variable with low conservation degree (Fig. 1b). Mutational results of W7Y have revealed that the −4 subsite played some of the roles in binding and catalysis, and once the binding affinity was changed, the mutation will inevitably affect the enzymatic activity. Thus, importance of the residues at relatively distant subsites also can’t be neglected.

With regard to the strength of hydrolytic interactions, it seems that tryptophan contributes to higher binding affinity than tyrosine, as the magnitude of the C-H…π interaction depends largely on the surface area^37^,^38^,^39^,^40^. However, we found that the binding constant of Tyr was larger than Trp. Virtual mutational analysis and theoretical calculations have revealed that additional hydrogen bonds may be formed by the hydroxyl group at a specific site of the glucosyl for the distance between cellohexose and the side chain of Tyr was below 5 Å. Formation of the new hydrogen bond may compensate for the loss of the hydrophobic area, providing a feasible explanation for the increase in binding affinity in both tyrosine substitutions, which were supported by other methods, such as ITC, Michaelis-Menten kinetics and FACE. As the configuration of amino acids depends largely on the microenvironment, such as its spatial position and side-chain properties, for aromatics, the balance between forming hydrophobic interactions and hydrogen bonds, and the relative strengths of the two interactions may be different^41^. This will ultimately result in various substrate-binding energies at different subsites^42^,^43^.

**Figure 6:**
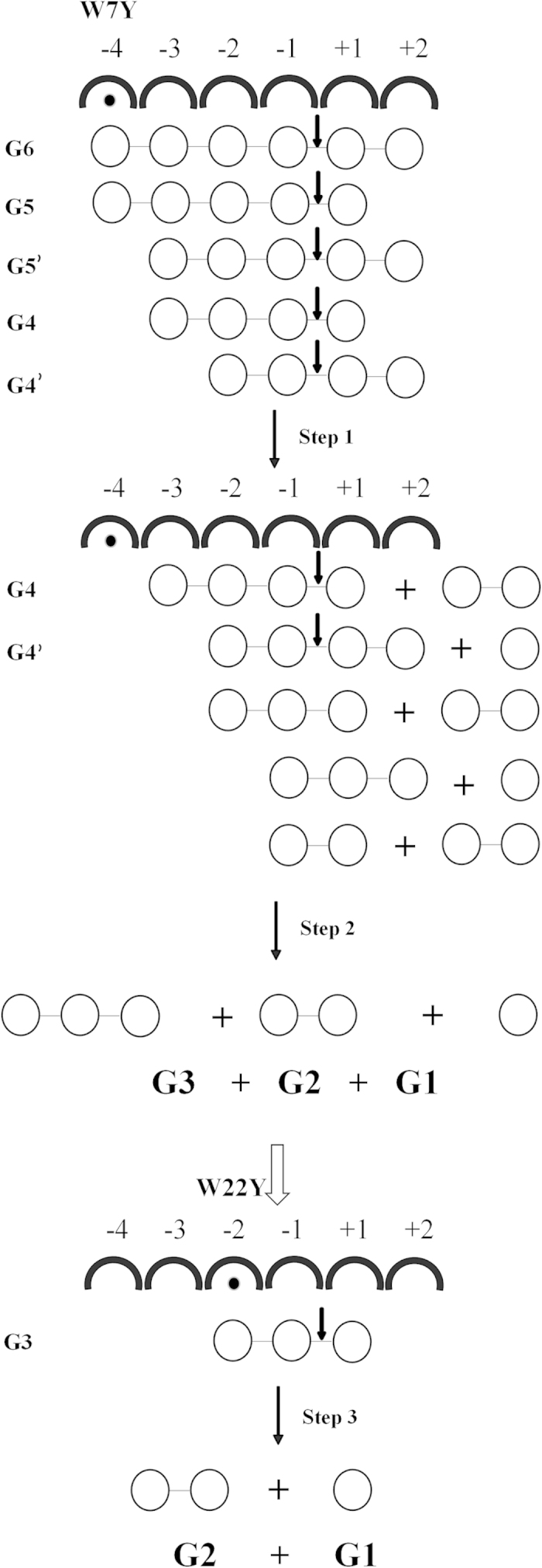
Schematic model of the influence of tyrosine on binding and catalysis. Substrates with different DPs are marked on the left of each step, while products in the ultimate are labeled below. Circles represent the glucose unit, while semicircles and the number above represent the active site and the corresponding glucose-binding subsites. The solid circle labeled at the −4 and −2 subsite indicates the site mutated. The bold arrow represents the catalytic site, while the spindly and the hollow one represent the reaction process and the functional transformation of enzymes with different modes of action.

In the present study, although the binding affinities of both W7Y and W22Y were enhanced, the endoglucanase activities changed dramatically (Fig. 2b), indicating that the roles and dynamics of the two different aromatics were distinct. In order to illustrate this discrepancy, a schematic model depicting the subsite-specific influence of tyrosine on binding and catalysis (Fig. 6) was proposed based on the product profiles shown by FACE. As both the binding energies at the subsites of −2 and −4 changed after mutation, the original binding-catalytic balance of the WT would no longer exist, thus new modes of action may be applied by W7Y and W22Y in recognizing the particular substructure of the heterogeneous amorphous cellulose. As there are six glucose-binding subsites in the large crevice of *Tr*Cel12A, the cellulose chain with DP > 6 spans the active site architecture and is then cut at the inner glycosidic bond, releasing a series of polysaccharides with lower DP values and ultimately cello-oligosaccharides as the reaction proceeds. Since G6 can exactly occupy the whole binding subsites, while G5 and G4 have two different binding patterns, respectively, the hydrolysate after the first step will compromise G1, G2, G3 and G4, and G4 mainly comes from G5 and G6. In the next step, G4, the minimum binding unit, was hydrolyzed into the final products G1, G2 and G3 (Fig. 6 Step 2). For W7Y, the enhanced binding affinity at the subsite of −4 mainly increased its binding capacity for G5 and G6 other than G5′, G4 or G4′ in the first step, which produced more G4 intermediate and provided more substrate accessibility in Step 2, leading to enhanced catalytic efficiency as shown in Fig. 2b and Table 1.

However, for the mutant of W22Y, increased binding energy at the subsite of −2 made it possible to bind G3, further expanding its substrate specificity (also known as “substrate promiscuity”) and leading to the final hydrolyzate of G1 and G2 as demonstrated in Fig. 5c and depicted in Fig. 6, Step 3. Previous studies have revealed that promiscuity was mainly due to partial recognition and binding to the molecular mimicry of the structurally repeated ligands through a small number of plasticity residues^44^. This discrepancy in the product profiles above further proved that W22 at the −2 subsite was the determinant of substrate specificity of the GH12 family, and a single substitution of the plasticity-determining residue was sufficient for the promiscuous to be more functionally divergent^45^,^46^. Transformation of the mode of action of W22Y to G3, similar to the signature of pseudo-processivity, further indicated that the key for oligosaccharide production is to improve binding interactions to stabilize the substrate in the active site architecture^47^. However, tighter binding at the −2 subsite hampered the smooth sliding movement of the product to a large extent (margin decrease in *k*_*cat*_ was larger than the increase in 1/K_m_ as shown in Table 1) and the resultant endoglucanase activity reflected by the amount of reducing sugar produced declined to 44.9% of the WT. This provides a reasonable explanation for the discrepancy in enzymatic efficiency resulting from mutations of the two specific binding subsites.

From the mutational analysis carried out in this study, it can be concluded that enzymes with “latent skills” are ideal alternatives for protein engineering, as it seems there is a tradeoff between substrate specificity and catalytic efficiency^45^,^46^. Higher specificity and improved activities could be achieved at the cost of less functionality. Also, residues with a lower degree of conservation in the active site architecture should receive further attention. Unlike the detrimental effect of the absolutely conservative residues, mutations in the less strict residues were amenable. Not only that, but it was possible to generalize an intrinsic quantitative structure-activity relationship (QSAR) by further analyzing the effects of the substitutions with similar side-chain properties. It will become easier to discriminate the specific contributions of different subsites to enzymatic functions and the characteristic modes of action of each GH family. In addition, binding capacity enhancement of the relatively distant residues should be emphasized to improve the catalytic efficiency of GH. Manipulations to extend the recognition sites to substrate, for example, by adding the non-catalytic carbohydrate binding module, was proved to be effective^48^. In addition, great success has been achieved in certain carbohydrate hydrolases^49^,^50^,^51^.

In conclusion, our comprehensive analysis of the GH12 endoglucanase provided a reasonable strategy for the rational design of GH. Enhancement of subsite-binding energy near the catalytic center will expand substrate specificity, while higher catalytic efficiency will be achieved by improving the binding affinities of the relatively distant subsites. It will be interesting to apply these findings in future protein engineering projects.

## Materials and Methods

### Bioinformatics data sources and construction of the sequence logo

Sequence information on the GH12 members with an EC number was retrieved from the databases of National Center for Biotechnology Information (NCBI, http://www.ncbi.nlm.nih.gov/), Uniprot (http://www.uniprot.org/) and CAZy, and structures of the resolved were downloaded from PDB. Multiple sequence alignments were built in ALN with the CLUSTAL algorithm using Mega v5.0 (http://www.megasoftware.net/) and the results were presented as a neighbor-joining tree built with bootstrap parameters in Newick. Taxonomy assignments were categorized with different colors by iTOL (http://itol.embl.de/).

To create a high-quality sequence profile of the active site architecture in the GH12 family, we combined the results of sequence and structure alignments. First, a new and reasonable cellulase-glucosyl complex of *Tr*Cel12A (member from *T. reesei*, PDB 1H8V) was simulated by superimposing the cellohexaose in *Bl*Cel12A (member from *Bacillus licheniformis*, PDB 2JEN) into the binding cleft of *Tr*Cel12A for only the co-crystal ligand of *Bl*Cel12A occupies all the six substrate-binding subsites and the two backbone structures were quite similar with a root-mean-square deviation (RMSD) of merely 0.874 Å. Then, *Tr*Cel12A was selected as the structural alignment model and pairwise alignment with the three-dimensional structures of other 10 members (PDB IDs 1W2U, 2BWC, 3VGI, 1KS3, 2JEN, 3VL8, 1OA3, 1OA4, 2NLR and 3AMP) to that of 1H8V was performed using PyMOL v1.5 software (http://pymol.org/dsc/ip/eula-r0211.html).

Residues with side chain atoms within a cut-off distance of 5 Å to the *Tr*Cel12A ligand were selected and corresponding sites in the other 10 PDBs were also mapped based on the structural alignment^52^. For the sequence-only members with an EC number, the corresponding residues were located through multiple sequence alignments and were further used together for generation of the active-site sequence logo of the GH12 family by WebLogo (http://weblogo.berkeley.edu/). Relative evolutionary conservation of each site, taking *Tr*Cel12A as the reference, was evaluated by the normalized ConSurf Score at the following website: http://consurf.tau.ac.il/index_proteins.php as described^53^.

### Gene clone, mutagenesis and protein purification

Wild-type (WT) *Tr*Cel12A (Egl3) with signal peptide excluded and the 6 ×His-tag designated at the C-terminal was cloned and inserted into pPIC9k (Invitrogen, Carlsbad, CA, USA), while mutants were constructed by site-directed mutagenesis according to the PCR-based method^54^. Competent yeast (*Pichia pastoris*) GS115 cells (Invitrogen) were then transformed with the recombinant plasmids after they were confirmed by DNA sequencing (Biosune, Shanghai, China). The exogenous proteins were induced and purified with the protocols described in the Original Pichia Expression Kit (Invitrogen) and the QIAexpress Kit (Qiagen, Hilden, Germany). All proteins expressed were buffer exchanged in 20 mM acetate buffer (pH 5.5) and the concentration was determined by the Bradford method^55^. Manipulations to another member of the GH12 family, *An*EglA, from *Aspergillus niger* CBS120.49 (PDB 1KS4) were the same to *Tr*Cel12A and the WT *An*EglA and its mutants were buffer exchanged in the same acetate buffer with pH 3.8. All chemicals, reagents and enzymes used were of analytical grade from Sigma (St. Louis, MO, USA) or Sangon (Shanghai, China).

### Circular dichroism

Circular dichroism (CD) spectra of the WT *Tr*Cel12A and its mutants were obtained in 20 mM acetate buffer (pH 5.5) using a CD spectrophotometer with the model of J-600 (Jasco, Tokyo, Japan) at 20 °C. All the proteins were concentrated to 0.2 mg/ml beforehand and data were averaged from three acquisitions. Scan rate was set at 10 nm/min with the wavelength of 190 nm to 260 nm.

### Reducing sugar assays

Enzymatic activities reflected by the reducing sugar produced were determined by the reaction at 50 °C in 20 mM acetate buffer and measured using a Spectra Max M5 microplate spectrophotometer (Bihe International Trading Limited, China) at the wavelength of 540 nm. The reaction mixtures of endoglucanase containing 600 μl of 2% (w/v) carboxymethylcellulose (CMC, Sigma) solution and 400 μl appropriately diluted enzyme samples were incubated for 30 min and assayed by the dinitrosalicylic acid (DNS) method^56^. Specific activities were defined as the amount of enzyme required for 1 μmol product/min and all measurements were conducted at least three times. Set the relative activity of the WT as 100%, other relative ones were calculated by dividing the corresponding specific activities by that of the WT.

### Isothermal titration calorimetry

Isothermal titration calorimetry was conducted with an ITC 200 calorimeter (MicroCal, Northampton, MA, USA). Titrations were performed by injecting 2 μl aliquots of different concentrations of the CMC solution into the ITC sample cell (volume of 200 μl) containing different enzyme samples at 25 °C. The stirring speed and reference power were set at 1000 rpm and 10 μcal/s, respectively. The heat background was measured under the same conditions by dropping the buffer only without ligand into the protein at the same concentration as in the cell. Data analysis was performed using ORIGIN 70 software (MicroCal) and thermodynamic parameters, such as the association constant (*K*_*a*_) and the binding enthalpy change (Δ*H*) were determined. Gibbs free energy change (Δ*G*) and the entropy change (Δ*S*) were calculated according to the following equations: Δ*G* = Δ*H*-TΔ*S* and Δ*G* = −RTln*K*_*a*_, where R and T represent the gas constant and the absolute temperature, respectively^57^.

### Theoretical calculations of interaction energy

The quantum mechanical calculations were carried out to determine the theoretical interaction energy (Δ*E*) of the glucose complex to indole, phenol, imidazole, benzene and methane (the side chain of tryptophan, tyrosine, histidine, phenylalanine and alanine, respectively) using the Gaussian09 program package (http://www.gaussian.com/g_prod/g09.htm). Full geometry optimizations without any symmetry constraints were performed within the framework of density functional theory (DFT) using the m06 functional with the standard 6–31G (d,p) basis set. Vibrational frequencies were also calculated at the same level of theory to confirm whether the optimized structures corresponded to a true local minima or not and to provide zero-point vibrational energies (ZPEs).

### Determination of kinetic parameters

To determine changes in kinetic parameters resulting from mutagenesis of the key residues, purified proteins (100 μl) of appropriate concentrations were kept in 500 μl CMC solution (20 mM acetate buffer, pH 5.5) at various concentrations (0.2–1.4%) at 50 °C for 5 min. Reactions were terminated by adding 400 μl DNS solution as described above and kinetic parameters, such as the turnover number (*k*_cat_), Michaelis constant (K_m_) and catalytic efficiency (*k*_cat_/K_m_) were nonlinearly fitted by the one site binding equation in GraphPad Prism 5 (GraphPad Software. Inc, USA).

### Time-course analysis of the hydrolytic pattern on amorphous cellulose

The hydrolysis product of *Tr*Cel12A and its mutants on amorphous cellulose, i.e. phosphoric acid-swollen cellulose (PASC) were analyzed by fluorophore-assisted carbohydrate electrophoresis (FACE). PASC was prepared as previously described^58^ and the concentration was determined by the phenol-H_2_SO_4_ method^59^. Mixtures of 1% (w/v) PASC solution and different enzyme samples (0.03 mg/ml) at a ratio of 1:1 (v/v) were kept at 50 °C for up to 2 h. Aliquots of 10 μl were taken out at specific time intervals and the enzymes were inactivated by boiling for 10 min. Fluorescent labeling using 7-amino-1,3-naphthalene disulfonic acid monopotassium salt monohydrate (ANDS) followed by electrophoresis using a Mini-PROTEAN 3 PowerPac Basic Power Supply (Bio-Rad, Hercules, CA, USA) were conducted as previously described^60^. Electrophoresis gels were photographed using a Chemidoc^TM^ MP system (Bio-Rad) at a wavelength of 302 nm and images were stored in the TIF format. The marker used was prepared in our laboratory according to a previously published method^61^ and treatment was the same as the samples described above.

## Additional Information

**How to cite this article**: Zhang, X. *et al.* Subsite-specific contributions of different aromatic residues in the active site architecture of glycoside hydrolase family 12. *Sci. Rep.* **5**, 18357; doi: 10.1038/srep18357 (2015).

## Supplementary Material

Supplementary Information

## Figures and Tables

**Table 1:** Kinetic parameters of *Tr*Cel12A and the tyrosine mutations to CMC.

Enzyme	K_m_(g/L)	*k*_cat_ (1/s)	*k*_cat_/K_m_ (L·g^−1^·s^−1^)
*Tr*Cel12A	22.74	277.8	12.21
*Tr*Cel12A-W7Y	12.05	168.3	13.96
*Tr*Cel12A-W22Y	13.16	72.06	5.48
