# Supplementary material for: Subsite-specific contributions of different aromatic residues in the active site architecture of glycoside hydrolase family 12
Source: Sci Rep. 2015 Dec 16;5:18357. doi: 10.1038/srep18357 (PMC4680936; doi:10.1038/srep18357)
Supplement: Supplementary Information [file srep18357-s1.pdf]

Subsite-specific contributions of different aromatic residues in the active site architecture of glycoside hydrolase family 12

Xiaomei Zhang[1], Shuai Wang[1], Xiuyun Wu[1], Shijia Liu[1], Dandan Li[1], Hao Xu[2], Peiji Gao[1], Guanjun Chen[1] & Lushan Wang[1,*]

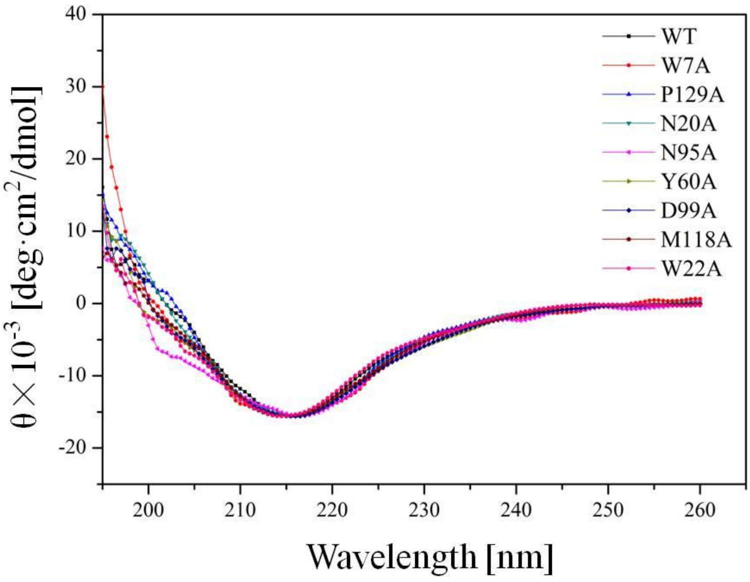

Figure S1 Circular dichroism spectra of the WT *Tr*Cel12A and its alanine mutants constructed.

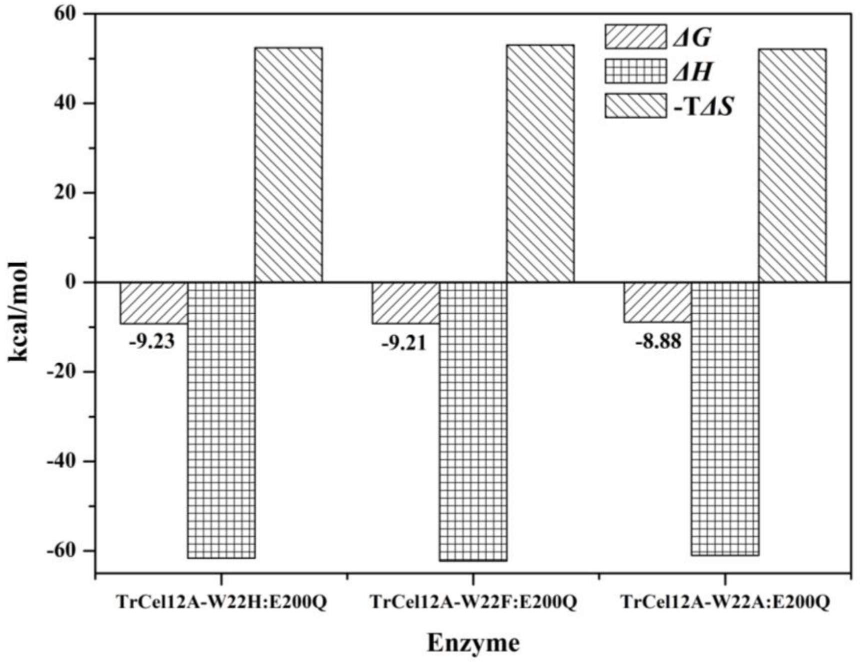

Figure S2 Comparison of the thermodynamic parameters determined for W22H, W22F and W22A. Concentration of *Tr*Cel12A-W22H:E200Q, *Tr*Cel12A-W22F:E200Q and *Tr*Cel12A-W22A:E200Q was 8.3 μM, 4.1 μM and 14.5 μM, and that of the corresponding ligand was 10.2 μM, 22.0 μM and 38.5 μM, respectively.

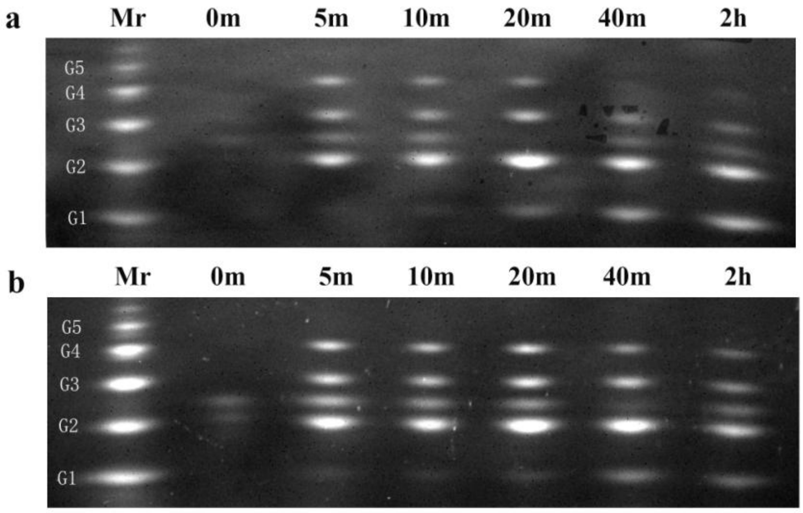

Figure S3 Time courses of the product profiles of *Tr*Cel12A-W22H (a) and *Tr*Cel12A-W22F (b) on PASC. The upper band next to G2 preexisted in the substrate has not been identified so far.

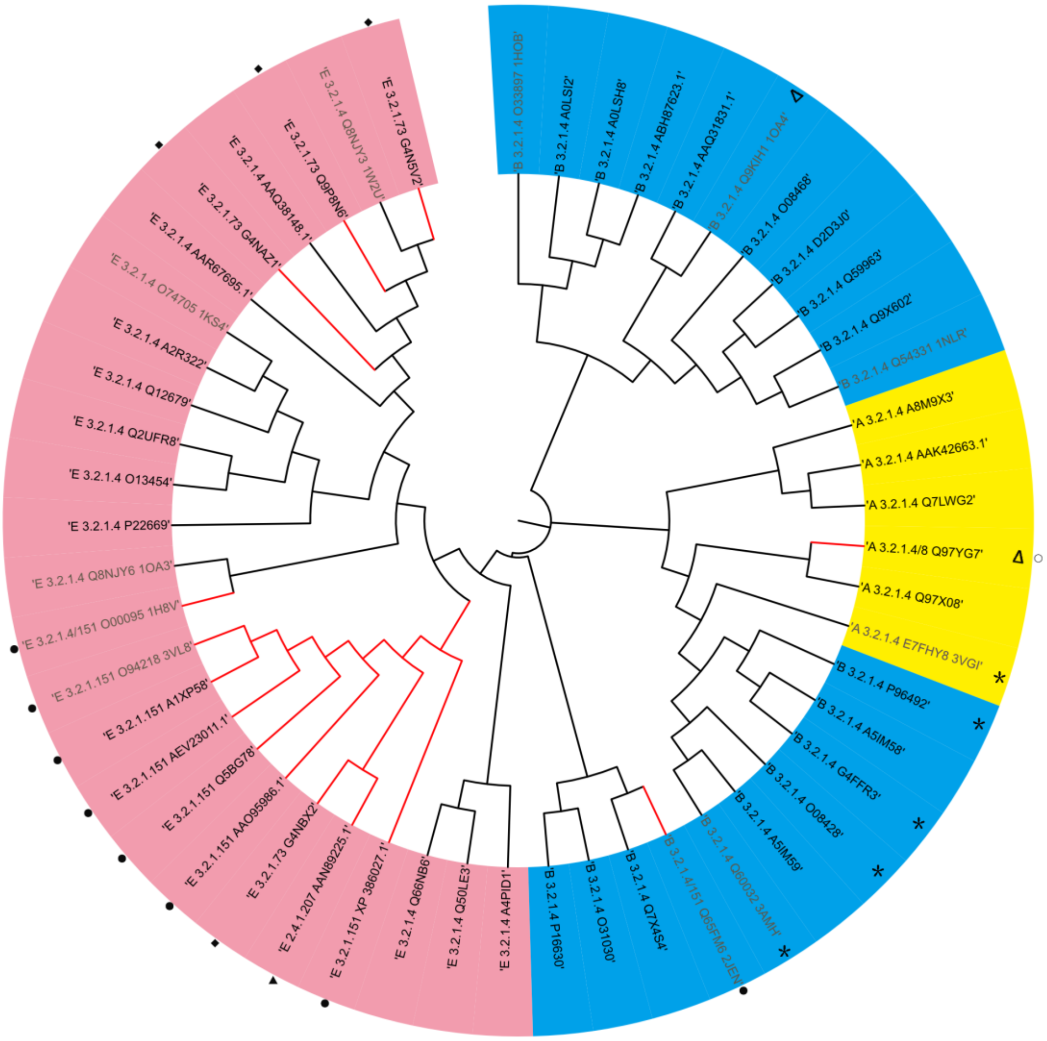

Figure S4 Phylogenetic tree of the GH12 family

Clusters and corresponding colors are: Archaea (A)—yellow, Bacteria (B)—blue and Eukaryota (E)—pink. Members with known activities, except endoglucanase (EC 3.2.1.4), are in the red branches and mapped with different symbols on the outer layer, where the solid circle (●), rhombus (■) and triangle (▲) represent xyloglucan hydrolase (EC 3.2.1.151), β-1,3-1,4-glucanase (EC 3.2.1.73) and xyloglucan endotransglycosylase (EC 2.4.1.207) and the hollow circle (○) represents xylanase (EC 3.2.1.8). Structure-resolved members are highlighted in gray and those with extreme pH_{opt} values (Uniprot No. Q97YG7 with pH_{opt} 1.8 from *sulfolobus solfataricus* and Q9KIH1 with pH_{opt} 8.0 from *Streptomyces* sp. 11AG8) and T_{opt} values above 95 ℃ (O08428 and P96492 from *Thermotoga neapolitana*; Q60032 and G4FFR3 from *Thermotoga maritima* and E7FHY8 from *Pyrococcus furiosus*) are labeled Δ and *.

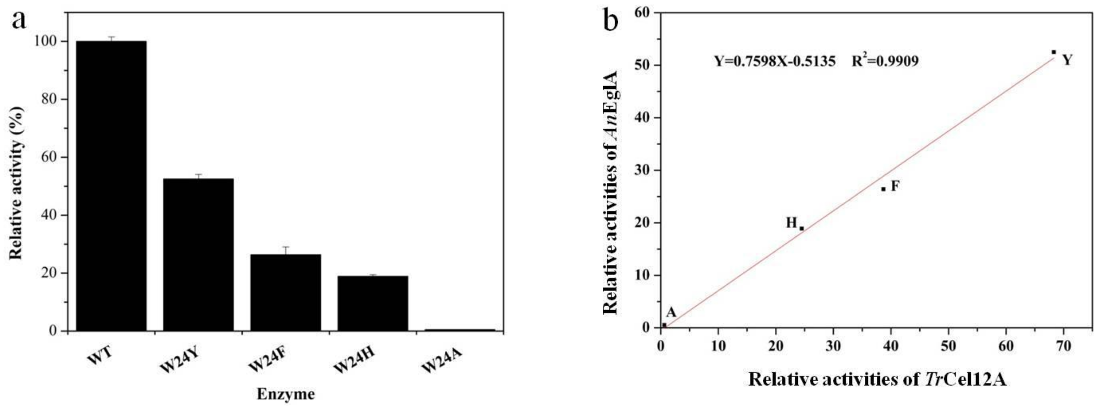

Figure S5 **Relative endoglucanase activities of *An*EglA and the mutants constructed on Trp24 at the -2 subsite (a) and the correlation of relative activities between *An*EglA and *Tr*Cel12A (b).**

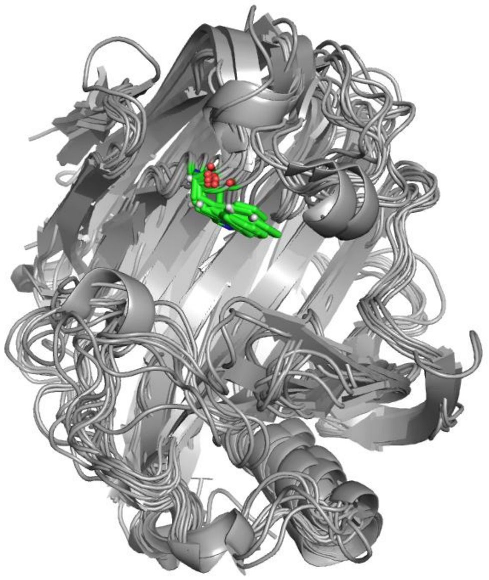

Figure S6 Superimposition of the GH12 members with PDB ID. Proteins were displayed in the cartoon pattern while the tryptophan at the -2 subsite highlighted in stick.

Table S1
Information to the known 3D structures in GH12 family*

Organism (Enzyme name)	PDB ID[a]	Catalytic residues[b]	RMSD(Å)[c]
Humicola grisea (Cel12A)	**1W2U**, 1OLR, 1UU4, 1UU5, 1UU6	120E, 205E	0.635
Trichoderma reesei (Cel12A/EGⅢ)	**1H8V**, 1OA2, 1OLQ	116E, 200E	_
Bacillus licheniformis ATCC 14580(Cel12A)	**2JEN**, 2JEM	155E, 243E	0.874
Aspergillus niger CBS120.49/N400 (EglA)	**1KS4**, 1KS5	116E, 204E	0.707
Hypocrea schweinitzii ATCC 66965 (Cel12A)	**1OA3**	116E, 200E	0.188
Streptomyces sp. 11AG8 (Cel12A)	**1OA4**	120E, 203E	1.014
Rhodothermus. marinus IT1378 (Cel12A)	**2BWC**, 1HOB, 2BW8, 2BWA, 2BWC, 3B7M	124E, 207E	1.104
Streptomyces lividans 1326（CelB）	**2NLR**, 1nlr	120E, 203E	0.894
Thermotoga maritima MSB8（CelA）	**3AMH**, 3AMM, 3AMN, 3AMP, 3AMQ, 3O7O, 3VHN, 3VHO	134E, 231E	3.845
Pyrococcus furiosus DSM 3638 （Cel12）	**3VGI**	197E, 290E	5.328
Aspergillus. aculeatus KSM 510 （XegⅠ）	**3VL8**, 3VL9, 3VLB	119E, 205E	0.597

*: Information was collected up to Jan 1st, 2015.

a: Those IDs in bold are the wild types.

b: Nucleophile and acid/base catalytic residues.

c: Root-mean-square deviation, denoted the average distance of the C-alpha atomic coordinates between each aligning molecule and 1H8V.

Table S2

Kinetic parameters of *Tr*Cel12A and the substitutions of Phenylalanine and Histidine at the -2 subsite

Enzyme	K_m (g/L)	k_{cat} (1/s)	k_{cat}/K_m (L g^{-1} s^{-1})
*Tr*Cel12A	22.74	277.8	12.21
*Tr*Cel12A-W22F	26.48	94.5	3.57
*Tr*Cel12A-W22H	43.20	137.9	3.19
